# A Deep Learning-Based Framework for Uncertainty Quantification in Medical Imaging Using the DropWeak Technique: An Empirical Study with Baresnet

**DOI:** 10.3390/diagnostics13040800

**Published:** 2023-02-20

**Authors:** Mehmet Akif Cifci

**Affiliations:** 1 The Institute of Computer Technology, Tu Wien University, 1040 Vienna, Austria; mcifci@tuwien.ac.at; 2 Department of Computer Eng., Bandirma Onyedi Eylul University, 10200 Balikesir, Turkey; 3 Department of Informatics, Klaipeda University, 92294 Klaipeda, Lithuania; meh.cifci@kvk.lt

**Keywords:** CT, lung cancer, deep learning, uncertainty quantification

## Abstract

Lung cancer is a leading cause of cancer-related deaths globally. Early detection is crucial for improving patient survival rates. Deep learning (DL) has shown promise in the medical field, but its accuracy must be evaluated, particularly in the context of lung cancer classification. In this study, we conducted uncertainty analysis on various frequently used DL architectures, including Baresnet, to assess the uncertainties in the classification results. This study focuses on the use of deep learning for the classification of lung cancer, which is a critical aspect of improving patient survival rates. The study evaluates the accuracy of various deep learning architectures, including Baresnet, and incorporates uncertainty quantification to assess the level of uncertainty in the classification results. The study presents a novel automatic tumor classification system for lung cancer based on CT images, which achieves a classification accuracy of 97.19% with an uncertainty quantification. The results demonstrate the potential of deep learning in lung cancer classification and highlight the importance of uncertainty quantification in improving the accuracy of classification results. This study’s novelty lies in the incorporation of uncertainty quantification in deep learning for lung cancer classification, which can lead to more reliable and accurate diagnoses in clinical settings.

## 1. Introduction

Lung cancer is a devastating disease that often has a grim prognosis. An early identification and diagnosis are vital to improving the chances of survival. One cutting-edge diagnostic tool for lung cancer is CT scan imaging. Deep learning (DL) has recently emerged as a powerful tool for processing medical images and has demonstrated great promise in detecting pulmonary nodules. However, the prediction accuracy of neural networks (NNs) in DL is uncertain, as the inner workings of their hidden layers are often considered a “black box,” making their predictions difficult to interpret [1]. Machine learning (ML) is also commonly used in real-world applications, but it also faces the challenge of hidden layers being considered as black boxes. Despite its potential for a high accuracy, DL is only sometimes reliable and can sometimes produce incorrect results [2]. It is essential to incorporate uncertainty into the models to increase the reliability of DL predictions and reduce the number of false predictions [3]. Additionally, DL models can be prone to overfitting and erratic behavior on out-of-distribution samples as they lack the reasoning capability to explain the data [4].

In the field of medical imaging, deep learning techniques such as convolutional neural networks (CNNs) and deep neural networks (DNNs) are widely used [5]. While CNNs can perform complex operations, they require a large, labeled dataset and strong processing power [6]. On the other hand, DNNs are a standard feedforward network but also need methods to estimate the uncertainty to enhance the reliability [7]. Quantifying uncertainty in deep learning (DL) is a difficult challenge, but Bayesian neural networks (BNNs) and Monte Carlo (MC) are effective methods for uncertainty quantification (UQ) [8]. Medical experts should be aware of the confidence level of DL models before they are used in various applications [9]. However, some DL models may produce unclear predictions because of their inability to handle noisy data or incorrect model inference [10].

Incorporating uncertainty into DL models can enhance their reliability and efficiency and help medical experts make more informed decisions. For instance, in diagnosing lung cancer, UQ can be used to identify areas in the images that require a further examination and avoid incorrect predictions. This is particularly crucial, as incorrect predictions can result in delayed treatment and decreased survival rates. Moreover, UQ can also be utilized to develop more robust and generalizable DL models that can adapt to new and unseen data. This is particularly important in the medical field, where there are often limited labeled datasets and new data types are constantly emerging [8].

In conclusion, uncertainty quantification in DL for medical imaging plays a crucial role in ensuring the reliability and efficiency of DL models and can lead to more informed decisions for medical experts.

Using uncertainty quantification in deep learning models can be crucial in the medical field, especially in diagnosing lung cancer. By incorporating uncertainty into these models, they can provide medical experts with a probabilistic interpretation of the predictions, which can help them understand the reasoning behind the model’s output. Bayesian deep learning methods, such as Bayesian neural networks (BNNs) and Monte Carlo dropout, have been shown to perform well in uncertainty quantification and provide more accurate and reliable results. These methods allow the model to randomly drop out neurons during the inference process, generating multiple predictions and helping to estimate the uncertainty in the model’s output. Incorporating uncertainty into deep learning models for medical imaging applications is crucial for improving the accuracy and reliability of the predictions. The use of Bayesian deep learning methods can provide medical experts with a better understanding of the reasoning behind the model’s output and help them make more informed decisions. Further research in this area can lead to the development of more robust and generalizable deep-learning models, which can significantly improve the accuracy of medical diagnoses.

Incorporating both aleatoric and epistemic uncertainty into the classification model can help improve the predictions’ reliability. The proposed method in this study considers both types of uncertainty by incorporating MC dropout into the classification model to estimate epistemic uncertainty and by using a Bayesian NN to estimate aleatoric uncertainty. The results of this study demonstrate the importance of considering uncertainty in medical imaging classification tasks as it can lead to the improved accuracy and reliability of the model’s predictions. It is important to note that the proposed method is only a starting point for further research in medical imaging classification and uncertainty quantification. There is still much work to be done to fully understand the impact of uncertainty on the predictions of deep learning models and to develop more robust and effective methods for incorporating uncertainty into these models. Nevertheless, this study highlights the potential of incorporating uncertainty into medical imaging classification tasks and the importance of continued research in this area.

**Figure 1:**
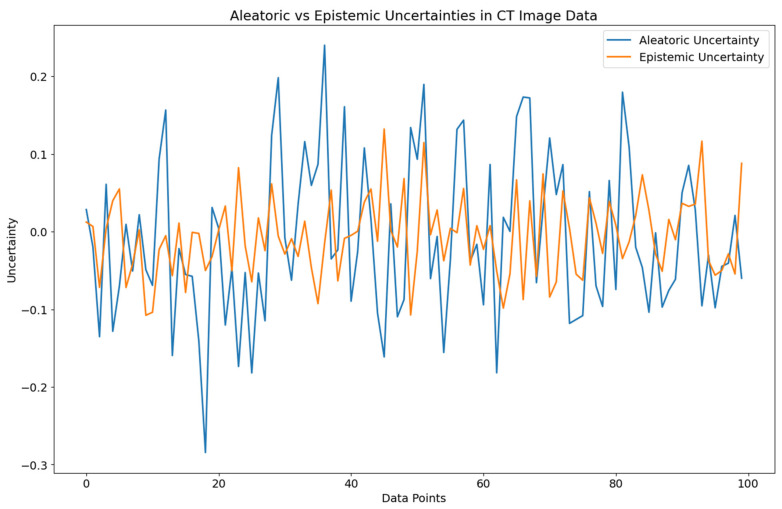
Aleatoric and epistemic uncertainties in the data.

Aleatoric uncertainty refers to the inherent uncertainty in the data, such as measurement errors or other sources of randomness. Epistemic uncertainty, on the other hand, refers to the uncertainty that arises from the model’s lack of knowledge about the genuine underlying relationship between the inputs and outputs. In medical imaging, aleatoric uncertainty could arise from measurement errors in the CT scan. In contrast, epistemic uncertainty arises due to the lack of knowledge or information about the data. These uncertainties can affect the accuracy and reliability of the predictions made by a model. In medical imaging applications, it is essential to consider and quantify both types of uncertainty to ensure the reliability and robustness of the model’s predictions. Figure 1 provides a visual representation of the two types of uncertainty and how they impact the model’s predictions. By incorporating uncertainty quantification techniques such as Bayesian deep learning or Monte Carlo dropout, the reliability and robustness of the predictions can be improved, making the models more useful for medical experts in decision-making.

In Figure 1, the orange line visually displays the uncertainty in the training data, while the blue line represents the uncertainty in the testing data, as seen in the CT scans of the lungs. This study aims to provide a unique deep learning model called Baresnet for automatically classifying lung cancer tumors using CT images. Baresnet is a cutting-edge system that blends the ResNet architecture with three standard models. The orange line represents aleatoric uncertainty, which arises from the intrinsic unpredictability or randomness in the data. Factors such as measurement errors, variability in the physical processes, or small sample sizes can cause this type of uncertainty. It cannot be reduced by gathering more data or improving the measurement procedures. The blue line represents the epistemic uncertainty resulting from insufficient knowledge or a lack of data-related information. This type of uncertainty can be due to various reasons, including a lack of data, uncertainty in the model parameters, or a poor understanding of the underlying processes. It can be reduced by increasing knowledge or obtaining better data and is often related to the accuracy of predictive models or algorithms. By visualizing both uncertainties in a single figure, it becomes easier to understand the sources of uncertainty in the data and to evaluate the effectiveness of various models and algorithms in reducing uncertainty.

In the context of lung cancer CT scans, aleatoric uncertainty can be caused by several factors, such as measurement errors during imaging, variability in the tissue density, or the limited sample sizes of CT scans. Epistemic uncertainty can result from a lack of information about the scans or the classification model. For example, suppose the model used for automated tumor classification is trained on various CT scans. In that case, it may not perform well on scans with different imaging characteristics, leading to higher epistemic uncertainty. A lack of understanding of the mechanisms contributing to tumor development can lead to a higher epistemic uncertainty. Baresnet aims to minimize both forms of uncertainty to increase the accuracy and reliability of lung cancer tumor classification. By merging ResNet with three standard models, we aim to incorporate stochastic and knowledge-based uncertainty to create more robust predictions. Minimizing uncertainty can provide medical professionals with more precise and reliable forecasts, leading to a better diagnosis and treatment decisions for lung cancer.

The purpose of this research is to address the lack of trust in the prediction of current data mining and image processing procedures for lung cancer detection. By introducing Baresnet, the researchers aim to achieve a state-of-the-art performance in tumor classification, improve the precision of the classification, and control feature selection using Grad-CAM. The proposed model achieved an accuracy of 97.19%, a precision of 91.68%, a sensitivity of 98.82%, a specificity of 92.56%, and an F1 score of 97.47% for lung cancer classification.

The key contributions of this work can be summarized as follows:

- Development of a novel method for automated lung cancer detection using CT scans: this work proposes a new approach for detecting lung cancer in CT scans that utilizes deep learning and computer vision techniques.
- Utilization of deep neural networks trained with Grad-CAM: the proposed approach utilizes deep neural networks trained with the Grad-CAM method, which helps to highlight the regions of the CT scan that contribute the most to the predictions made by the model.
- Achieving a state-of-the-art performance in tumor classification: the proposed approach has been shown to achieve a state-of-the-art performance in terms of the accuracy and precision in tumor classification, outperforming the existing methods in the field.
- Improving the precision and control over the feature selection with Grad-CAM: by utilizing the Grad-CAM method, this work also improves the precision of the classification and provides a mechanism for controlling feature selection in the model, which is essential for ensuring the robustness of the results.

The main difference between our paper and the state of the art.

The text discusses the importance of uncertainty quantification (UQ) in deep learning (DL) for medical imaging. DL techniques, such as CNNs and DNNs, are widely used in medical imaging, but their hidden layers are often considered a “black box,” making their predictions difficult to interpret. Incorporating a UQ can improve the reliability and efficiency of DL models, allowing medical experts to make more informed decisions. Bayesian neural networks (BNNs) and Monte Carlo (MC) are effective methods for UQ in DL. By considering uncertainty, DL models can be adapted to new and unseen data, making them more robust and generalizable.

## 2. Related Studies

Diverse DL methods have dominated the world of medical image processing. Be-yond automatic radiological functions (e.g.; segmentation, detection, disease grading, and classification), ML has been applied to various “data enhancement” problems. Data processing or optimization seeks to improve accuracy, primarily due to the widespread implementation of DL methods in many fields. BNN and UQ predictions have gained more interest [11]. BNN approaches reflect uncertainty by putting a probability distribution over the model parameters and adding up these probabilities to construct a final uncertainty estimate. In the late 1990s, the state-of-the-art approach to training NN was Brown et al.’s work [12]. As demonstrated in [13], many parameters are required for predictive modeling, making these approaches repetitive. It is challenging to incorporate BNN approaches into contemporary NN [14]. For many decades, MC was the gold standard for inference with NN, thanks to the work of Hamiltonian MC. In Ref. [15], the Hamiltonian MC did not have ample tuning possibilities and would be unfeasible for current NNs. A stochastic gradient Hamiltonian MC (SGHMC) approach was developed, which is a method for estimating the posterior distribution of the parameters in a Bayesian neural network (BNN) [16]. SGHMC is a combination of Hamiltonian Monte Carlo (HMC) and stochastic gradient descent (SGD) methods. In HMC, the posterior distribution is estimated using Hamiltonian dynamics and gradient information, while in SGD, gradient information is used to minimize the loss function. In SGHMC, the gradient information is used to guide the sampling process in HMC, resulting in more efficient and accurate posterior estimates. SGHMC has been shown to be effective in BNNs with large and complex models, where the posterior distribution is highly non-Gaussian [16]. Alternatively, stochastic gradient Langevin dynamics [17] can be used by first-order dynamics in the stochastic gradient setting. Asymptotically, SGHMC and stochastic gradient Langevin dynamics sample from the posterior distributions of σw for each model specification on an infinitely small phase scale. Using a limited sample rate might make it more challenging to combine approximations with tweaking [18]. Moreover, accurately estimating the uncertainty of NNs is a significant challenge. However, various methods have assessed uncertainty, such as predictive entropy, shared knowledge, and an average estimate of multiple models [19]. BNN helps model uncertainty by providing a natural context for computations. Furthermore, BNN models offer a straightforward way to model the degree of uncertainty in a case’s probability that might provide various possible out-comes. Various approximations have been developed, and multiple methods can be used, including the Markov chain approximation (MCA) and stochastic gradient approximations, which is another method for estimating the posterior distribution in BNNs. MCA approximates the posterior distribution as a Markov chain, with each iteration of the chain representing a sample from the posterior distribution [20]. MCA can be used in combination with other methods, such as Monte Carlo methods, to improve the accuracy of the posterior estimates. MCA is computationally efficient and can be applied to large and complex models, making it a popular choice for BNNs [21]. The Hamiltonian approximations in [22] are attributable to multiplicative normalizing flows, stochastic batch normalization, maximal softmax approximations, and qualified confidence estimations, including deep ensembles to fit NNs into BNN. In some instances, deterministic networks’ weight parameters are replaced with the prior distribution of the same parameters, and then the networks’ weights are optimized directly. Although this basic model works reasonably well, the assumptions are not as good Although this fundamental model functions well, the underlying assumptions are not as sound for more complex problems. The basic model assumes, for instance, that the prior distribution over the parameters is a simple Gaussian distribution, which may not be acceptable for complex, high-dimensional parameter spaces. Batch normalization is a technique used to improve the training and generalization of deep neural networks. It was introduced by Sergey Ioffe and Christian Szegedy in their paper “Batch Normalization: Accelerating Deep Network Training by Reducing Internal Covariate Shift” in 2015 [23]. The technique works by normalizing the inputs to each layer of the network, reducing the internal covariate shift. The internal covariate shift refers to the change in the distribution of the activations within the network during the training process. Batch normalization helps reduce this shift by normalizing the activations for each mini batch during training, which can help stabilize the training process and improve the overall accuracy of the model.

Various techniques have been proposed in medical image classification to detect tuberculosis (TB) in chest X-rays (CXRs). [24] presents a wavelet transform-based approach as an alternative to conventional handcrafted feature extraction methods. The authors gather line profiles from CXRs and apply a one-dimensional discrete wavelet transform to obtain Daubechies coefficients, which are then used as features for TB identification.

In Ref. [25], an automated method for detecting TB in CXRs using texture patterns is described. The lung fields are divided into sections and evaluated individually. Multi-scale filter banks extract different texture features, such as the second, third, and fourth moments. The k nearest neighbors (K-NN) method is then employed to categorize texture patterns ranging from average (0) to abnormal (1).

Ref. [26] proposed using a histogram of oriented gradients (HOG), Gabor, and gist features for TB diagnosis in CXRs without segmentation. The results showed that the extracted features outperformed the gray-level co-occurrence matrix (GLCM) textural features in discriminating between TB and non-TB CXR images. In Ref. [27], a collection of feature extraction algorithms (e.g.; shape and texture features) were created using a wrapper-based feature selection approach to distinguish between normal and TB CXR lung images. The authors obtained an accuracy of 78.3% and an area under the curve of 0.87 for the Montgomery dataset, an accuracy of 95.57%, and an AUC of 0.99 for the Shenzhen dataset.

In Ref. [28], deep learning-based approaches were employed to classify X-ray images for TB detection using an ensemble of three pre-trained CNNs (ResNet50, VGG19, and InceptionV3). The images were preprocessed by horizontal mirroring and applying histogram equalization or CLAHE.

Ref. [29] utilized an ensemble of finely tuned CNNs to classify medical images from the subfigure classification dataset of the ImageCLEF 2016 collection. The authors create a novel feature extractor by fine-tuning the CNN architectures AlexNet and GoogleNet, combined with SoftMax and one-vs-one multi-class SVM classifiers.

Ref. [30] suggested using lung area symmetry to identify pulmonary problems. Abnormal posteroanterior chest radiographs (CXRs) are likely to show changes in the lung content (textures), size, and shape, which are examined using edge plus texture features and multi-scale shape features. The classification architecture is a blend of multilayer perception neural networks (MLP), Bayesian networks, and random forests based on a voting system. The approach has a detection accuracy of 91.0% and an AUC of 0.96, based on data collections as seen in Table 1.

## 3. Materials and Methods

This section aims to provide a comprehensive overview of a lung cancer diagnosis method, its underlying techniques and procedures, as well as the recent advancements and challenges in this field. The primary objective is to evaluate the potential of using uncertainty quantification (UQ) in the context of medical image analysis, particularly for the purpose of enhancing the reliability of the diagnostic model.

### 3.1. Image Dataset

The study focuses on the use of Bayesian deep learning methods, specifically Baresnet, for the diagnosis of lung cancer. The dataset used in the study was obtained from LIDC-IDRI, a publicly available database that contains 244,527 images of 1010 cases. The images were obtained from clinical thoracic CT scans performed by four experienced thoracic radiologists.

The images were annotated through a two-phase image annotation procedure and were available in various formats, including scalable vector graphics for screen-reading devices such as cell phones or tablets and XML for display on a computer or printer. The images showed extensive thickening of the lung nodules, most of which were concentrated at 1, 1.25, and 2.5 mm. After pre-processing, the sizes of the pulmonary nodules were expected to range from 3 mm to 30 mm, with a larger number of benign nodules having a small diameter and a smaller number of malignant nodules having a larger diameter.

The XML commentary file for each patient made it possible to locate and assess the degree of malignancy in pulmonary nodules. Four radiologists reviewed the pulmonary nodules in the XML format and classified the degree of malignancy into five categories: highly unlikely for cancer, moderately unlikely for cancer, indeterminate likelihood, moderately suspicious for cancer, and highly suspicious of cancer. The first two categories were classified as non-malignant, while the last two categories were classified as malignant.

Table 2 provides important information about the LIDC-IDRI dataset for lung cancer detection and diagnosis. Specifically, it outlines the categories used by the four experienced thoracic radiologists to classify the degree of malignancy in pulmonary nodules, as well as other key features of the dataset.

Table 2 shows that the degree of malignancy was classified into five categories, ranging from “highly unlikely for cancer” to “highly suspicious of cancer”. The first two categories were classified as non-malignant, while the last two categories were classified as malignant. 

### 3.2. MC Dropweak in Convolutional Neural Networks

In the field of medical image classification, convolutional neural networks (CNNs) have demonstrated remarkable success. However, a common challenge in training these models is the risk of overfitting, particularly with imbalanced datasets. To mitigate this issue, various techniques have been developed, including random sampling and L2 regularization [40]. One such technique is DropOut, introduced by Hinton et al. in 2012, which is a regularization method that prevents overfitting by randomly dropping out neurons during the forward pass of each training iteration. This random dropout of neurons has the effect of making the network more robust and less sensitive to individual neuron weights, reducing an overfitting risk [41]. The dropped-out neurons are reactivated in the next training iteration and the process is repeated until the end of training. Another regularization technique is DropConnect, introduced by Wan et al. in 2013, which operates at the weight level rather than the neuron level. DropConnect randomly zeroes out individual weights in the network instead of dropping out entire neurons, reducing their contribution to the final prediction. The dropped weights can be reactivated in the next training iteration, as in DropOut [42]. In this study, we employ MC Dropweak, a combination of DropOut and DropConnect, to address the issue of overfitting. MC Dropweak operates by randomly dropping out neurons and dropping individual weights, making the network more robust and reducing the risk of overfitting. Additionally, MC Dropweak resets any weights with low values to zero, as these are typically associated with noisy inputs and do not contribute to accurate predictions. The dropped weights and neurons can be reactivated during training if they are deemed essential to the accuracy of the predictions. MC DropWeak has been shown to provide better results than either DropOut or DropConnect alone.

**Figure 2:**
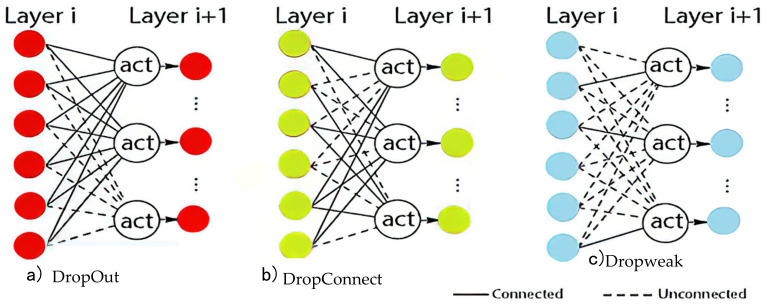
Comparison of (**a**) DropOut; (**b**) DropConnect and (**c**) MC Dropweak node activation.

Figure 2 shows that the dropout nodes become ineffective. Meanwhile, DropConnect extends the functionality of DropOut and MC Dropweak combines both by allowing the nodes to be inactive and activatable as required.

In conclusion, the paper describes different techniques for preventing overfitting in convolutional neural networks (CNNs) for medical image classification. It introduces DropOut, a regularization method that randomly drops out neurons during the forward pass of each training iteration, making the network more robust and less sensitive to individual neuron weights. DropConnect is another regularization technique that operates at the weight level by randomly zeroing out individual weights, reducing their contribution to the final prediction. The text then describes MC DropWeak, which combines DropOut and DropConnect, and has been shown to provide better results than either method alone by making the network more robust and reducing the risk of overfitting. The dropped weights and neurons can be reactivated during training if deemed essential to the accuracy of the predictions.

### 3.3. Bayesian Neural Networks

The Bayesian classifier is a probabilistic method of classification that calculates the threshold parameters systematically through computation, as opposed to relying on a heuristically determined rule. It categorizes a pixel, x, as belonging to the sputum region if the probability of it being background (p(bg|x)) is lower than the probability of it being sputum (p(sp|x)). While traditional deep learning (DL) techniques have shown success in various real-world problems, they lack the ability to provide a measure of their predictions’ reliability. To address this issue, we utilized a Bayesian neural network (BNN) in our study. BNNs are models that incorporate probabilistic reasoning and represent random variables as independently and identically distributed inputs and one-hot encoded categorical outputs. BNNs can also identify uncertainties in classification problems and generate probabilistic predictions by computing complex mathematical computations. There are two types of uncertainty, aleatoric and epistemic, and the BNN model is formulated by considering the posterior distribution *p(X, Y)* through the prior distribution, likelihood, and posterior steps, such as building an alpha-level Bayesian belief network (BBN) model. The posterior distribution provides information about the uncertain quantities in the Bayesian analysis as seen in Equation (1).

(1) px^,X,Y=∫Px^,wPX,Y^dw

The equation in question models the joint probability distribution of three variables: “*x^* “, “*X*”, and “*Y^*”. It represents the probability of observing these variables simultaneously, taking into account the relationship between them and a fourth variable “*w*”. The joint probability distribution is calculated as an integral over all the possible values of “*w*”. The integrand, *P(x^,w)P(X,Y^)*, is the product of two probability distributions. The first, *P(x^,w)*, represents the joint probability of observing the variables “*x^*” and “*w*”, while the second, *P(X,Y^)*, represents the joint probability of observing the variables “*X*” and “*Y^*”. The result of the integration is the joint probability distribution of all three variables “*x^*”, “*X*”, and “*Y^*”, considering the relationship between these variables and the variable “*w*”. This relationship between the variables can be interpreted as a form of uncertainty present in the system, which is represented by the variable “*w*”. The mutual information present in *p(x^,X,Y)* is taken into account in the calculation of the joint probability distribution. It is important to note that averaging the predictions from an ensemble of neural networks based on the posterior distribution results in the same outcome as the distribution *p(w|X, Y)*. In other words, using an ensemble of neural networks allows us to incorporate the uncertainty present in the system and improve the accuracy of our predictions. This serves as a measure of bias-corrected epistemic uncertainty, reflecting the heterogeneity in the weight configurations predicted by the NNs, which depend on the estimated posteriors. This approach extracts an approximation of the finite bias from the population through entropy leave-one-out estimators, leading to a significant reduction in bias. The leave-one-out estimator is a resampling method used to evaluate the performance of a model by iteratively leaving out one sample and training the model on the remaining data. The prediction for the left-out sample is then compared with the actual value to compute the error. This process is repeated for each sample in the dataset and the errors are aggregated to obtain a measure of the model’s performance. In the context of Bayesian neural networks, the leave-one-out estimator can be used to estimate the finite bias present in the population by removing one sample at a time and computing the entropy of the predicted probabilities. This provides an approximation of the population’s bias, which can be used to reduce the overall bias in the predictions made by the NNs.

### 3.4. Proposed Baresnet Model

This study aims to address the challenge of overfitting in medical image classification by utilizing transfer learning on a small dataset of CT scans. Specifically, the study fine-tunes a pre-trained ResNet-based Baresnet model, which includes a fully connected layer on top of the base ResNet layer and MC DropWeak for model uncertainty estimation. The study uses a combination of the Naïve Bayes classifier and ResNet to create Baresnet. The Naïve Bayes classifier is a straightforward probabilistic classifier that performs well with categorical variables, while ResNet is a deep learning neural network architecture for “Residual Networks.” The key innovation behind ResNet is the use of residual connections, which allow information to bypass multiple layers in the network and prevent vanishing gradients. The study also highlights the importance of the learning rate, a critical hyperparameter that determines the speed and convergence of the model. The environment for training these models typically involves a computing system with a GPU for acceleration and a large dataset of medical images, along with corresponding labels, for training and testing purposes.

The environment for training these models would typically involve a computing system with a GPU for acceleration and a large dataset of medical images, along with corresponding labels, for training and testing purposes. The learning rate is a crucial hyperparameter that determines the speed and convergence of the model. It is often set through trial and error and can significantly impact the model’s performance. To prevent overfitting, the study split the CT scan dataset into training (80%), testing (10%), and validation (10%) sets and performed real-time data augmentation. The images were rescaled to 256 × 256 pixels and standardized using the mean and standard deviation of the dataset. The study used the Adam optimizer with a learning rate of 1 × 10^−5^ and a factor of 0.2 and set the batch size to 16. The experiment was run for 750 epochs, and the validation accuracy was assessed after each epoch.

MC DropWeak was applied during training and Baresnet conducted Monte Carlo (MC) sampling by feeding the input image MC samples of 10, 25, and 50. MC DropWeak was added to the outputs of the FCL, allowing the model to dynamically change the drop probability per weight. The performance activation formula was expressed as follows in Equation (2).

(2) $$\sum_{M}^{f}\left(\left(\left(M,⊙,W\right),x\right)\right)\approx f\left(\sum_{M}^{f},\left(\left(M,⊙,W\right),x\right)\right)$$

The equation is related to the Monte Carlo method. The Monte Carlo method is a statistical method that uses random sampling to estimate a function’s value or solve a problem. In the context of the equation provided, the matrices “*M*” and “*W*” are used to generate multiple random realizations of some underlying process, and the function “*f*” is used to map these realizations to some output. The equation then approximates the output of “*f*” by taking the sum of the product of the matrices “*M*” and “*W*” for each realization and applying the function “*f*” to the result. The approximation in the equation is based on the law of large numbers, which states that the average of a large number of independent random variables converges to their expected value. The approximation assumes that the sum of the product of the matrices “*M*” and “*W*” for each realization represents a large number of independent random variables and that the result of the sum is close to their expected value.

**Figure 3:**
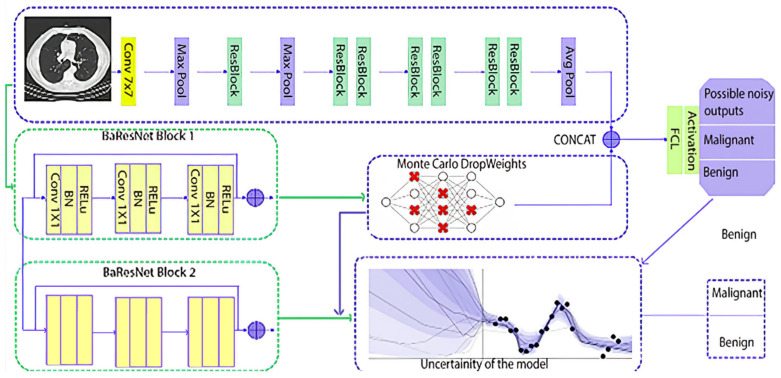
Proposed Baresnet model architecture: A study on uncertainty quantification in lung cancer diagnosis.

The *M*-bit binary string is mapped to the truth table representation of the function. The p-bit mask encodes the connection information drawn from a Bernoulli distribution with probability *p*. The weight between the *j*_th_ neuron in layer l-1 and the *i*_th_ neuron in layer l is represented by $W_{ij}^{\left(l\right)}$. The drop probability of the weight associated with $W_{ij}^{\left(l\right)}$ being set to 0 is represented by $\rho_{ij}^{\left(I\right)}$. The proposed Baresnet model is depicted in Figure 3, displaying the architecture of the model.

The Bernoulli distribution is a fundamental concept in probability theory that has widespread applications in various fields, including statistics, machine learning, and data science. In its simplest form, the Bernoulli distribution models the probability of success and failure in a binary event, where success is represented by a value of 1 and failure is represented by a value of 0. The Bernoulli distribution is defined by a single parameter, *p*, which represents the probability of success. The probability of failure is simple (1 − *p*).

The study demonstrates the effectiveness of transfer learning and MC DropWeak in addressing the challenge of overfitting in medical image classification. The study also highlights the importance of hyperparameters, such as the learning rate and the use of real-time data augmentation to prevent overfitting. The study provides insights into the use of ResNet and Baresnet architectures for medical image classification and their applications in the automated classification of lung cancer tumors using CT images. DropOut, DropConnect, and MC DropWeak are regularization techniques used to mitigate overfitting in CNNs. In medical image classification, MC DropWeak has been shown to provide better results than either DropOut or DropConnect alone. ResNet and Baresnet are deep learning architectures used in medical image classification, particularly for the automated classification of lung cancer tumors using CT images. The SoftMax layer generated the classmark probability distribution.

Figure 3 depicts the architectural configuration of a neural network, which consists of two components: Baresnet Block 1 and Baresnet Block 2. Baresnet Block 1 is directly connected to MC Dropweak, while Baresnet Block 2 is connected to UQ. The MC Dropweak technique is applied to the first block, as only one block is required to implement MC Dropweak. On the other hand, the second block is placed directly into the model, without undergoing any pre-processing step. Furthermore, Block 2 is subjected only to the DropOut regularization technique.

#### Uncertainty Quantification

Uncertainty quantification (UQ) [43] is an important aspect of machine learning (ML) applications, particularly in medical imaging. In the field of lung cancer, the use of UQ in combination with ML models can provide valuable information about the degree of certainty in diagnosis and treatment decisions.

In ML, it is common to distinguish between two types of uncertainty: epistemic and aleatoric. Epistemic uncertainty refers to the uncertainty in the model parameters, which can be reduced with more training data. Aleatoric uncertainty, also known as data uncertainty, captures the observation noise and cannot be reduced with more data collection, but may be reduced with a more accurate sensor output. CT scans, commonly used in the diagnosis of lung cancer, have an epistemic degree of uncertainty that can be characterized using alternative probability distributions for discrete random variables and probability density functions for continuous variables.

Data used in ML models are often small, incomplete, noisy, and multimodal, leading to a high degree of uncertainty in predictions made without proper UQ analysis. To achieve reliable results in DL, it is important to use the most robust and varied databases available, and to use UQ to understand the limitations of the model and the data used. The DL models used in lung cancer diagnosis and treatment, such as Baresnet, are structured to achieve specific performance targets, and the teaching process is replicated with new learning conditions to optimize the performance.

In conclusion, UQ is essential in ML applications in lung cancer, providing valuable information about the degree of certainty in diagnosis and treatment decisions. By characterizing and quantifying uncertainty in the data and the models used, we can improve the reliability and accuracy of ML-based predictions in lung cancer.

In the context of lung cancer, CT scans are commonly used for diagnosis and can have significant amounts of uncertainty associated with them. This uncertainty can stem from both the data itself and the models used to analyze the data. For instance, the CT scans may be noisy or of a low resolution, and the models used may not be well-calibrated or may not have seen similar data during training.

By incorporating UQ techniques, we can account for both aleatoric and epistemic uncertainty in the CT scans and models. For example, probabilistic models can be used to represent aleatoric uncertainty by capturing the inherent noise in the observations. Meanwhile, Bayesian models can be used to represent epistemic uncertainty by characterizing our uncertainty about the model parameters.

Additionally, UQ can also play a crucial role in model selection and validation. By quantifying uncertainty in the model predictions, we can better understand the reliability of different models and choose the one that is best suited for the task at hand. This can also help us identify cases where additional data or improved models are needed to achieve accurate predictions.

Overall, the use of UQ in ML applications for lung cancer can help improve the accuracy and reliability of diagnosis and treatment decisions. By taking uncertainty into account, we can develop more robust and trustworthy ML-based tools for lung cancer diagnosis and treatment.

## 4. Experimental Results

In this study, we applied various methods to quantify the uncertainty in the predictive models used for lung cancer diagnosis and treatment decisions. These methods included the use of a predictive uncertainty estimator which measured the average standard deviation of the class probabilities for each class. However, this approach was not sufficient as it did not capture the full extent of uncertainty in the classification. To overcome this limitation, we introduced Bayesian methods for the estimation of uncertainty, using MC Dropweak during both the training and testing steps.

Additionally, we studied two methods for measuring uncertainty in classification tasks: a tractable, dynamic model of mutual information (MI) and a bias-corrected model of MI. MI is a well-known measure of uncertainty that tests the difference between a prediction and the posterior distribution of a model’s parameters. We used the entropy of the predictive distribution as an uncertainty metric to compare the results of our approach with those of other competitors.

Moreover, the optimization process not only helps to improve the accuracy of the predictions but also provides valuable information about the confidence in the predictions. This information is crucial in the context of medical applications as it can help to guide diagnosis and treatment decisions. In conclusion, incorporating UQ in the training process of the Baresnet model for lung cancer detection allows for a better representation of the inherent uncertainty in the data and the models, resulting in an improved reliability and accuracy of ML-based predictions.

Overall, the uncertainty estimation of the Baresnet model’s predictions is the sum of two terms: the true mean of the system and the uncertainty caused by the uncertainty in the process and the error in the model itself. This highlights the importance of incorporating UQ in ML applications for lung cancer, as it provides valuable information about the degree of certainty in diagnosis and treatment decisions and helps to improve the reliability and accuracy of ML-based predictions, as seen in Equation (3).

(3) $$H_{a}\left(y_{1},\ldots y_{n}|x_{1},\ldots x_{n}\right)=E_{q\left(w|\theta \right)}\left[H\left(y_{1},\ldots y_{n}|x_{1},\ldots x_{n},w\right)\right]$$

Equation (3) seeks to optimize the mean reciprocal knowledge between the prediction made by the model and its posterior distribution. Essentially, it represents the amount of information gained about the model’s parameters by observing the model’s predictions for a given sample. The mutual information (MI) between the model’s output (*y*) and its parameters (*w*) is calculated using the model’s epistemic uncertainty. The first term of the equation represents the entropy of the model’s predictions, which is critical when the predictions are inaccurate. The second term estimates the comparison between the entropy of the model’s predictions and the estimated posterior, taking into consideration the model’s parameters. This term, however, is unreliable in reflecting the model’s ability to account for the uncertainty associated with predictions made based on the estimated posteriors and weight configurations.

**Figure 4:**
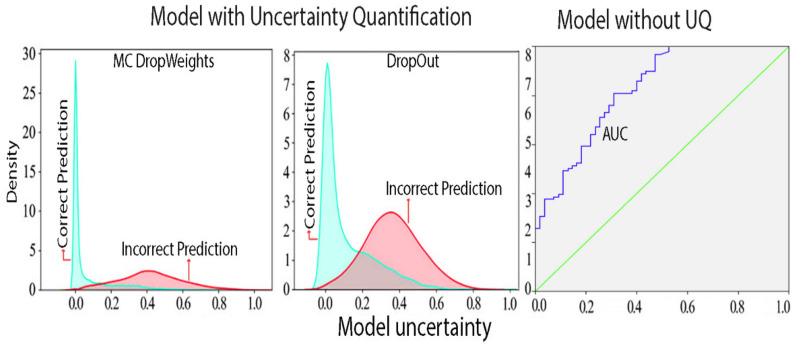
Comparison of Baresnet model’s predictive uncertainty with and without MC Dropweak.

Figure 4 illustrates the distribution of the potential values, which is influenced by the sample size and demonstrates significant levels of bias.

As illustrated in Table 3, the Baresnet model demonstrated impressive results in its performance metrics. The average sensitivity was recorded at 91.32%, the precision was 87.38%, the accuracy was 90.44%, the specificity was 89.59%, and the F1-score was 87.88%. The best results were achieved through k-fold validation, where the highest scores were obtained in the sensitivity (98.82%), precision (91.68%), accuracy (97.19%), specificity (92.56%), and F1-score (97.47%). The data in Table 3 highlights the effectiveness of the Baresnet model in accurately classifying lung CT scans.

**Figure 5:**
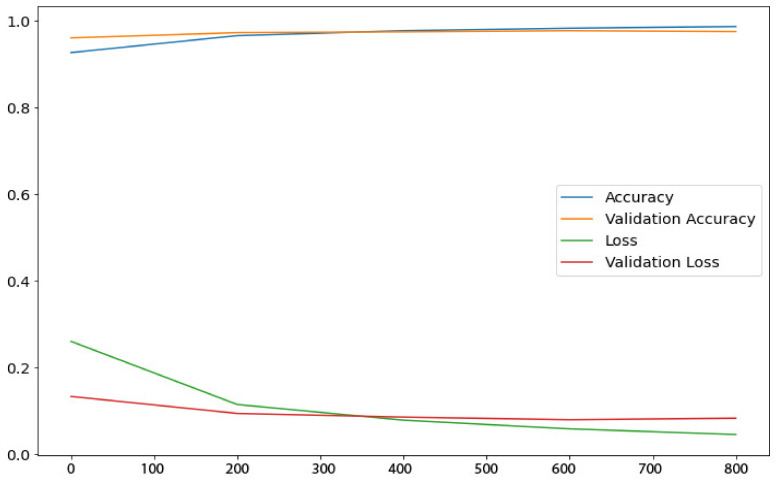
Performance evaluation of the proposed model: accuracy and loss values.

To incorporate uncertainty quantification (UQ) in the Baresnet model, we employed MC Dropout and a Bayesian neural network (BNN). The results of the model’s loss and accuracy are visualized in Figure 5. This step was crucial to complete the classification research, as reporting predicted uncertainty estimations is important in this domain.

In Figure 5, the performance of the proposed model is demonstrated through the presentation of its accuracy and loss values. The blue curve represents the loss values, while the orange curve displays the accuracy of the model. The analysis was conducted on all CT images, and the predictions were sorted based on their corresponding PH values. PH values, also known as the probability of hit, are commonly used in the field of signal processing and machine learning to evaluate the accuracy of binary classifier models. The PH value represents the proportion of positive predictions (hits) that are correct among all the positive predictions made by the model.

**Figure 6:**
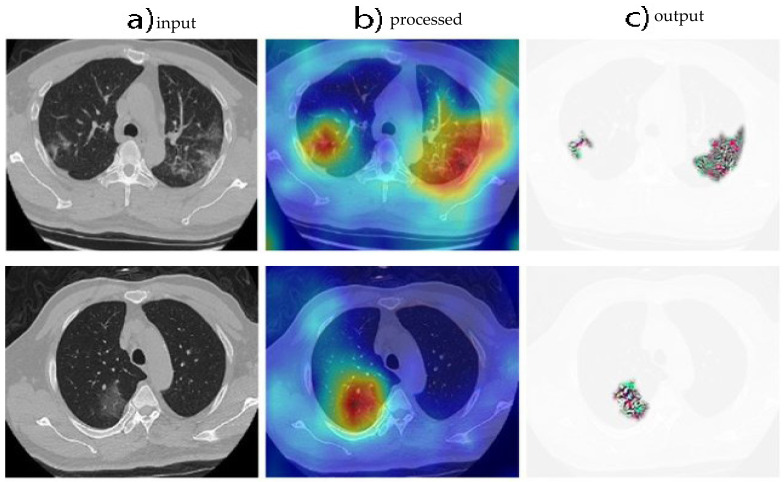
Grad-CAM visualization of model predictions on CT images.

In the context of the study presented in Figure 5 and Figure 6, PH values were used to sort the predictions made by the model on the CT images. By analyzing the predictions based on their associated PH values, the researchers were able to evaluate the model’s performance and calculate the accuracy threshold. The ROC curve in Figure 6 provides a visual representation of the model’s ability to distinguish between positive and negative predictions, and the AUC value quantifies the overall accuracy of the model in a single numerical value. It is important to note that PH values can be used in conjunction with other performance metrics, such as the sensitivity, precision, and F1-score, to gain a comprehensive understanding of a model’s accuracy and performance.

The predictions were then evaluated for their uncertainty levels, and the accuracy threshold was calculated for values of 0.3 and 0.5.

Figure 6 provides further insight into the performance of the proposed model through the depiction of the receiver operating characteristic (ROC) curve. The area under the curve (AUC) value is 97.19, indicating a strong correlation between the level of uncertainty in the model’s predictions and its accuracy. This result highlights the effectiveness of the proposed model in accurately classifying lung CT scans.

In Figure 6, three visualizations are presented to further demonstrate the performance of the proposed model. Figure 6a showcases the original CT image used in the analysis. Figure 6b provides a representation of the Grad-CAM map, which was generated using Monte Carlo Dropweak. Grad-CAM is a technique used to visualize the regions of the image that contribute most to the model’s predictions. The Grad-CAM map highlights the regions of the image that have the highest impact on the model’s decisions. Finally, Figure 6c displays the high-resolution attentional regions generated using Baresnet and Monte Carlo Dropweak. These regions provide a detailed view of the model’s focus and attention when making predictions and allow researchers to better understand the reasoning behind the model’s decisions. Together, these visualizations provide a valuable insight into the inner workings of the proposed model and how it processes and analyzes CT images to make predictions.

**Figure 7:**
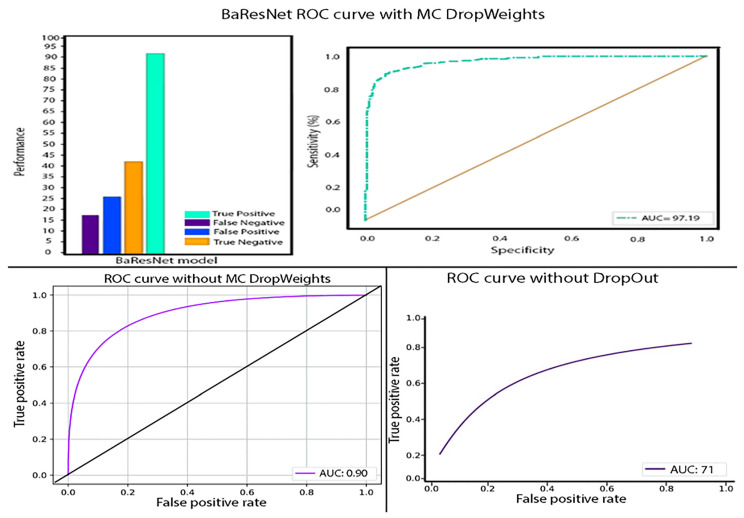
Baresnet model ROC curve and accuracy rates.

Figure 7 displays the performance of the Baresnet model in the form of a receiver operating characteristic (ROC) curve, widely used to assess the effectiveness of classification models. The high accuracy of the model is evident from the curve, which shows an area under the curve (AUC) of 97.19. This implies that as the model’s true positive rate increases, so does the accuracy of its predictions.

Moreover, Figure 7 also visualizes the level of uncertainty in the model’s predictions using a heat map. The darker colors on the map correspond to higher uncertainty levels, indicating that the model faced difficulty arriving at a consensus on the predicted label for samples located in regions of high color intensity. This indicates that the model experienced high predictive uncertainty in such instances.

### Distribution of Uncertainty Estimates

**Figure 8:**
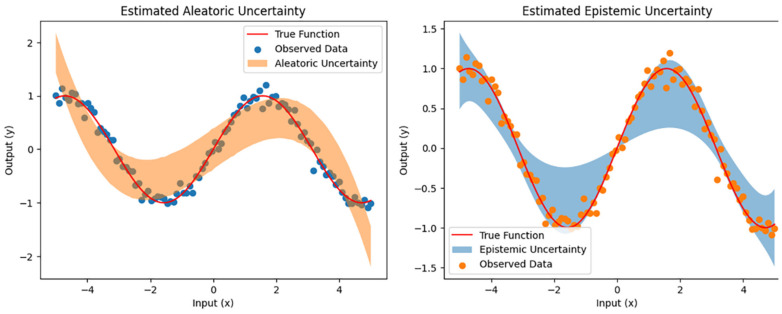
Distribution of estimated aleatoric uncertainty.

As displayed in Figure 8, the distribution of the AUC appears to be multimodal, meaning that there are multiple peaks or modes present. These modes are seen to be close to 0.24 units, which indicates that the distribution is not uniform.

Multimodality in the distribution of AUC is often the result of incorrect classifications. This can arise from the presence of irreducible homoscedastic and heteroscedastic noise in the data, which refers to random fluctuations in the measurement that cannot be reduced through repeated measurements. This type of noise can lead to inaccuracies in the model’s predictions, causing the distribution of the AUC to be multimodal. As a result, it is important to identify and address the sources of such noise to improve the performance and accuracy of the model.

Figure 8 provides a clear representation of the distribution of the estimated aleatoric uncertainty in the predictions made by the Baresnet model. The regularity of the epistemic uncertainty distribution and the concentration of correct predictions at the lower end of the uncertainty scale suggest that the model is performing well in terms of uncertainty estimation.

Table 4 provides a comparison of the results obtained by various state-of-the-art methods applied to several datasets. The results are presented in terms of sensitivity and precision (or AUC). The first column of the table lists the authors of the studies, and the second column lists the datasets used in each study. The third column of the table lists the methods used to analyze the data, including 3D DL, V-Net architecture, VGG16, ResNet50, CNN, Gaussian blur, Otsu thresholding, watershed transform, 2D CNN, RCNN, deep belief network, Boltzmann machine, extreme learning machine, and deep transfer. The fourth column lists the sensitivity and precision (or AUC) results obtained by each method.

The results show that Baresnet with the MC Dropweak method achieved a sensitivity of 98.8 percent, surpassing other typical ML techniques, such as 2D CNN and RCNN, CNN, deep belief network, Boltzmann machine, extreme learning machine, and deep transfer, which demonstrated lower sensitivity results ranging from 82.2 to 97.9 percent. The results also indicate that Baresnet with the MC Dropweak method is more effective than other typical ML techniques and outperforms the other state-of-the-art methods, including those that use V-Net architecture, VGG16, ResNet50, Gaussian blur, and Otsu thresholding.

## 5. Conclusions and Future Work

In this study, a modified version of Baresnet was used for the detection of lung cancer in CT scan images. The model was designed to provide two forms of quantifiable uncertainties, which were aleatoric uncertainty and epistemic uncertainty. The results revealed a strong correlation between the model uncertainty and prediction accuracy, indicating the significance of incorporating uncertainty quantification techniques.

Comparing Baresnet with other existing studies and popular deep learning methods, we found that Baresnet outperformed other techniques, especially when uncertainty quantification was incorporated. Our study highlighted the potential of the novel Baresnet architecture with uncertainty quantification techniques in improving the accuracy and diversity of the data samples for lung cancer detection.

It is important to note that the use of traditional uncertainty estimation techniques is limited in accurately estimating uncertainty in lung segmentation, and our study demonstrates the advantages of using Baresnet with uncertainty quantification techniques in improving the reliability and trustworthiness of the predictions. As a limitation, it should be noted that in this study, we only tested our modified Baresnet model with one dataset for lung cancer classification. Although we have demonstrated promising results, the performance of the model on other datasets may vary. Future studies could investigate the generalizability of the model with different datasets and further validate the effectiveness of the uncertainty quantification techniques.

In conclusion, this study demonstrates the potential of Baresnet with uncertainty quantification techniques in improving the accuracy of lung cancer detection. Future work will explore other model architectures to further improve the quality of epistemic uncertainty estimates. The findings of this study have significant implications for the clinical use of deep learning in lung cancer detection and can contribute to better patient outcomes.

## Figures and Tables

**Table 1:** Performance Metrics for Various Machine Learning Models in Lung Cancer Detection and Classification.

Method	Dataset	Accuracy	Reference
3D CNN unsupervised learning model	LUNA	Ineffective (10% training)	Moitra and Mandal (2020) [31]
Supervised CNN predictor	Real-time non-SCLC patient data	71% AUC (Insufficient)	Yu et al. (2020) [32]
3D-CNN	LIDC-IDRI, LUNA 16,	91.12%	Polat and Danaei Mehr (2019) [33]
DenseNet model	201 lung scans	90.85%	Fathalla et al. (2022) [34]
Deep Learning	Histopathological pictures	96.33%	Masud et al. (2021) [35]
CNN-RNN hybrid network model for EGFR mutation status evaluation	LIDC-IDRI	94.78%	Lin et al. (2022) [36]
Computer-aided diagnosis support system for lung nodule diagnosis (3D-DCNN)	LUNA16, ANODE09,	88.21%	Wang et al. (2020) [37]
Binary particle swarm optimization with decision tree (BPSO-DT) and CNN for cancer tissue detection	Super Bowl Dataset 2016	76.73% (insufficient)	Kasinathan at al. (2022) [38]
Tobacco exposure pattern (TEP) classification model	Two independent LUAD datasets	94.65% (training), 91.85% (validation)	Ryu et al. (2021) [39]

**Table 2:** Characteristics and Features of the LIDC-IDRI Dataset for Lung Cancer Detection and Diagnosis.

Field	Description
Number of cases	1010
Number of images	244,527
Image format	Scalable vector graphics (for screen-reading devices such as cell phones or tablets) and XML (for display on a computer)
Image focus	Thickening of lung nodules
Nodule size	3 mm to 30 mm
Annotation	Two-phase image annotation procedure by four experienced thoracic radiologists
Categories	5 (highly unlikely for cancer, moderately unlikely for cancer, indeterminate likelihood, moderately suspicious for cancer, highly suspicious of cancer)
Total images collected	910

**Table 3:** Evaluation of Baresnet model performance metrics.

Fold	Sensitivity	Precision	Accuracy	Specificity	F1-Score
*K-fold_1*	90.47	88.43	88.78	89.57	91.15
*K-fold_2*	84.56	89.29	88.57	87.49	89.92
*K-fold_3*	95.28	90.15	91.41	85.88	84.56
*K-fold_4*	98.82	91.68	97.19	92.56	97.47
*K-fold_5*	82.13	80.46	91.78	85.98	85.16
*K-fold_6*	94.38	89.44	96.55	89.47	98.28
*K-fold_7*	89.97	82.89	87.38	94.86	82.74
*K-fold_8*	89.75	85.69	86.94	90.67	80.34
*K-fold_9*	96.56	88.47	85.36	89.85	81.38
Average	91.32	87.38	90.44	89.59	87.88

**Table 4:** Comparison to the state of the art with 4K-ESA.

References	Datasets	Method	Result (%)
Bhattacharyya et al. (2022) [44]	LUNA 16	3D DL, V-Net architecture	Sensitivity: 96.5
Al-Shabi et al. (2019) [45]	ACDC LUNGH	VGG16, ResNet50, CNN	Sensitivity: 97.9 Specificity: 93
Chaturvedi et al. (2021) [46]	LUNA-16	Deep Learning	dice coefficient: 88.89%
Gwenzi et al. (2021) [47]	UCI	Gaussian blur, Otsu thresholding	Sensitivity: 87 Specificity: 97
Yu et al. (2021) [48]	LUNA16	3D and 2D CNN	Precision: 87 Specificity: 99.1
Shen et al. (2022) [49]	LoDoFanB	NeRP	AUC: 0.89
Pan et al. (2019) [50]	LIDC-IDRI	CNN, deep belief network, Boltzmann machine	Sensitivity: 82.2 AUC: 81.8
Huang et al. (2020) [51]	LIDC-IDRI	CNN, extreme learning machine and deep transfer	Sensitivity: 91.6 Specificity: 86.5
4K-ESA	NCA	Baresnet	Sensitivity: 98.8 Specificity: 97.1

## Data Availability

Not applicable.
